# Complete genome sequence of a novel *Prescottella* sp. R16 isolate from deep-sea sediments in the western Pacific

**DOI:** 10.3389/fgene.2024.1356956

**Published:** 2024-03-14

**Authors:** Lingqi Ma, Yuqi Bai, Weili Wang, Shengxiang Pei, Gaiyun Zhang

**Affiliations:** ^1^ Key Laboratory of Marine Biogenetic Resources, Third Institute of Oceanography, Ministry of Natural Resources, Xiamen, Fujian, China; ^2^ Department of Environmental Sciences, College of the Coast & Environment, Louisiana State University, Baton Rouge, LA, United States; ^3^ State Key Laboratory of Marine Environmental Science, College of the Ocean and Earth Science, Xiamen University, Xiamen, Fujian, China; ^4^ Hainan Observation and Research Station of Ecological Environment and Fishery Resource in Yazhou Bay, Hainan Institute of Zhejiang University, Sanya, Hainan, China

**Keywords:** *Prescottella*, whole-genome sequencing, phylogenetic analysis, biotechnological applications, extreme environments

## Abstract

*Prescottella*, a distinct genus separate from *Rhodococcus*, has garnered attention for its adaptability and ecological versatility. In this study, a Gram-stain positive and ovoid-rod shaped the actinobacterium strain R16 was isolated from deep-sea sediment (with a depth of 6,310 m) in the Western Pacific. On the basis of 16S rRNA gene sequence analysis, average nucleotide identity and phylogenomic analysis, strain R16 clearly represents a novel species within the genus *Prescottella*. Genomic analyses indicate *Prescottella* sp. R16 contains a circular chromosome of 4,531,251 bp with an average GC content of 68.9%, 4,208 protein-coding genes, 51 tRNA genes, and 12 rRNA operons. Additionally, four CRISPRs and 24 genomic islands are also identified. The presence of rich categories related to catalytic activity, membrane part and metabolic process highlights their involvement in cellular component, biological process, and molecular function. The genome sequence of strain R16 also revealed the presence of 13 putative biosynthetic gene clusters for secondary metabolites, including those for ε-Poly-L-lysine, ectoine, heterobactin, isorenieratene and corynecin, suggesting its potential for antibiotic production and warranting further exploration.

## 1 Introduction

The emergence of *Prescottella* as a distinct genus, separate from *Rhodococcus*, has drawn attention due to its adaptability and ecological versatility (Paterson et al., 2019; Kalinowski et al., 2020). Phylogenetically distinct from other rhodococci, strains of *Prescottella* sp. were reclassified as a new genus through comprehensive taxonomic analysis (Jones et al., 2013; Sangal et al., 2022). *Prescottella* sp. exhibits extraordinary resilience in extreme environments, thriving in diverse settings such as soil, water, and plant ecosystems. Its remarkable adaptability is further demonstrated by its isolation from varied environmental sources, including animal manure and rock cores (Kalinowski et al., 2020; Ivshina et al., 2022). In biotechnology, *Prescottella* exhibits significant potential for synthesizing valuable compounds, including biosurfactants and bioflocculants (Cappelletti et al., 2020). It also plays a crucial role in biodegradation (Ivshina et al., 2022) and bioremediation (Kuyukina and Ivshina, 2010). Notably, it excels in degrading pollutants in contaminated ecosystems and transforming pharmaceutical compounds. Its role in biocatalysis, especially in the development of pharmaceutical precursors and new drugs, underscores its importance (Anteneh and Franco, 2019; Busch et al., 2019).

Recent advancements in Whole-genome sequencing (WGS), particularly with third-generation sequencing using PacBio technology, have revolutionized bacterial functional analysis. A complete and highly accurate genome sequence serves as a pivotal foundation for unraveling the genetic features of microorganisms. In this context, high-throughput sequencing technologies, including third-generation sequencing, have played a critical role in obtaining such genome sequences (Didelot et al., 2012; Ma et al., 2016). Particularly, PacBio technology offers an advanced microbial genome assembly method characterized by extended read lengths and reduced GC bias.

In this study, we report the high-quality sequence of a novel strain of *Prescottella* sp. R16 strain, isolated from marine sediment at a depth of 6,310 m in the Western Pacific. This sequencing was conducted using the Single Molecule Real-Time (SMRT) technique on the PacBio Sequel platform. After assembly, recycling, error correction and annotation, a complete genome map of 4.53 Mb (including 1 plasmid) was obtained. Further bioinformatics analysis revealed its potential in lipid transport, metabolism, and amino acid biosynthesis. The complete genome sequence will provide valuable insight for its application in biotechnological and natural product biosynthesis applications.

## 2 Materials and methods

### 2.1 Bacterial strain isolation and cultivation

The bacterial strain was obtained from deep-sea sediment samples collected from the western Pacific Ocean, located at coordinates N10°54.7′ and E142°19.9′, at a depth of 6,310 m. Isolation was performed by spreading the sediment samples on a specialized isolation medium containing 10 g of glucose, 5 g of peptone, 5 g of yeast extract, 0.2 g of MgSO_4_·7H_2_O, 10 g of NaHCO_3_, 27 g of Na_2_CO_3_·10H_2_O, and 20 g of agar per liter of natural seawater with a pH of 10. Incubation took place at 28°C for a duration of 3 weeks. A single bacterial colony was selected and subsequently streaked onto plates containing marine agar 2216 (MA; Becton Dickinson) to obtain pure cultures. These cultures were cryopreserved at −80°C in a glycerol suspension comprising 15% (v/v) glycerol and 0.5% (w/v) trehalose.

### 2.2 16S rRNA sequence amplification and analysis

Genomic DNA extraction was carried out using a bacterial genomic DNA extraction kit (TaKaRa, Dalian, China). The near-complete 16S rRNA gene of strain R16 was amplified from the genomic DNA and sequenced using universal bacterial primers 27F (5′-AGA​GTT​TGA​TCC​TGG​CTC​AG-3′) and 1492R (5′-GGT​TAC​CTT​GTT​ACG​ACT​T-3′). The sequence was compiled using Contig Express software and compared with 16S rRNA gene sequences of valid species from GenBank via the BLAST program and the EzTaxon-e server (Yoon et al., 2017).

### 2.3 Morophological, physiological and biochemical characteristics

Cell morphology of strain R16 was observed with light microscopy after incubation on MA medium at 25°C for 3 days. Standard Gram staining was performed as described by Cerny (1978). Growth was tested at different temperatures (10, 15, 20, 25, 30, 35, 40°C and 45°C) and pH (4.0–12.0, at intervals of 1.0 pH unit) on MA liquid medium. NaCl tolerance (0%–8%, at intervals of 1%, w/v) was also tested using salt-free MA liquid medium as the basal medium. The growth of the strains was observed at 8 h intervals, in extreme conditions, the observation time should be extended to 14 days. Catalase activity was detected by the production of bubbles after the addition of a drop of 3% (v/v) H_2_O_2_. Oxidase activity was determined by the oxidation of tetramethyl-*p*-phenylenediamine. H_2_S production test and hydrolysis of cellulose and starch were performed as described by Li et al. (2007). Additional physiological and biochemical characteristics of strain R16 were carried out using API ZYM (bioMérieux) and API 20NE (bioMérieux) kits according to the manufacturer^’^s instructions.

### 2.4 Whole-genome sequencing and annotation

The complete genome of the bacterial strain R16 was sequenced utilizing the PacBio Sequel system (Pacific Biosciences, Menlo Park, CA, USA). Genome assembly was performed following a hierarchical genome-assembly process (HGAP) as described by Chin et al. (2013) using HGAP4 (v. 6.0) software. Open-reading frame (ORF) prediction was accomplished using Prodigal (v. 2.6.3) software with default parameters, and ORFs spanning sequencing gap regions were excluded. The bacterial proteome was annotated using BLAST (v. 2.6.0) software by alignment with the Clusters of Orthologous Groups (COGs) database. Metabolic pathways within the bacterium were reconstructed using the online tool KEGG Mapper (Kanehisa and Sato, 2020). Identification of ribosomal RNA (rRNA) genes and transfer RNA (tRNA) genes was performed using RNAmmer (v. 1.2) and tRNAScanSE (v. 2.0.9) (Lagesen et al., 2007), respectively. Clustered regularly interspaced short palindromic repeats (CRISPR) elements were identified using CRISPRCasFinder (v. 4.3.2) with default parameters (Couvin et al., 2018). Genomic islands were detected using the web tool IslandViewer (v. 4.0) with the independent methods Islander and IslandPath-DIMOB with default parameters (Bertelli et al., 2017). The presence of secondary metabolic gene clusters was assessed using the antiSMASH 5.0 platform with default parameters (Blin et al., 2019). The pan-genome analysis was performed on the IPGA platform v1.09 (https://nmdc.cn/ipga/ (Liu et al., 2022). The parameters of the analysis process are all default values. CheckM (v.1.0.18) was used to assess the completeness and contamination of the reference genomes that used in this paper based on marker gene sets of 111 essential single-copy genes. Average nuclear identities (ANI) and 16S rRNA similarity were evaluated between ten reference genomes utilizing pyani (https://github.com/widdowquinn/pyani) and Blastn with the initial setting.

### 2.5 Phylogenomic analysis

Utilizing whole-genome sequences represents a promising approach for elucidating the phylogenetic relationships among microorganisms. When it comes to strain identification, the gold standard in evolutionary phylogeny analysis relies on the examination of core genomes, which is more robust than relying on a single gene marker or concatenated sequences of a limited number of genes. With this rationale in mind, we conducted a phylogenomic analysis based on the core genes found in the entirety of the genome sequences of ten typical strains, all of which exhibited genome completeness exceeding 95%. These genome sequences were retrieved from the National Center for Biotechnology Information (NCBI) database.

The extraction of core genes was carried out using the state-of-the-art Bacterial Core Gene (UBCG) pipeline (Na et al., 2018). Subsequently, these genes were concatenated, and a maximum-likelihood tree was constructed employing the Genetic Testing Registry (GTR) model, facilitated by the RAxML (v. 7.0.4) tool (Stamatakis, 2014). The selection of 92 core genes was informed by a comprehensive dataset comprising 1,429 complete genome sequences spanning 28 phyla, ensuring the inclusion of genes that are either widely distributed across genomes or exhibit high conservation as single-copy genes.

## 3 Results and discussion

### 3.1 Physiological and biochemical analysis of R16 strain

Colonies of strain R16 grown on MA medium were observed to be circular, opaque, smooth and slightly convex, with light pink colouration after incubation for 3 days at 25°C. The cells of the organism were found to be Gram-stain positive, ovoid-rod shaped, occurring singly or in pairs. Growth of strain R16 occurred at 20°C–35°C, pH 5-9 and in 0%–4% (w/v) NaCl, with optimal growth occurring at 25°C, pH 7 and in 1% NaCl. Oxidase-negative and catalase-positive. Negative for H_2_S production and hydrolysis of cellulose and starch. In the API ZYM strip, positive for the activity of alkaline phosphatase, lipase (C14), cystine arylamidase, trypsin, *α*-chymotrypsin, *α*-glucosidase, *N*-acetyl-*β*-glucosaminidase, *β*-glucuronidase, *β*-glucosidase, *α*-galactosidase, *β*-galactosidase, *α*-mannosidase and *β*-fucosidase; negative for the activity of esterase (C4), leucine arylamidase, acid phosphatase, naphthol-AS-BI-phosphohydrolase, esterase lipase (C8) and valine arylamidase. In the API 20NE strip, positive for reduction of nitrate to nitrite, urease activity and hydrolysis of *p*-nitrophenyl-*β*-D-galactopyranoside and esculin; negative for denitrification, indole production, glucose fermentation, arginine dihydrolase activity, hydrolysis of aesculin and gelatin, and assimilation of adipic acid, D-glucose, D-mannose, D-maltose, L-arabinose, *N*-acetylglucosamine, D-mannitol, potassium gluconate, capric acid, malic acid, trisodium citrate and phenylacetic acid.

### 3.2 Phylogenetic analysis of R16 strains

**FIGURE 1:**
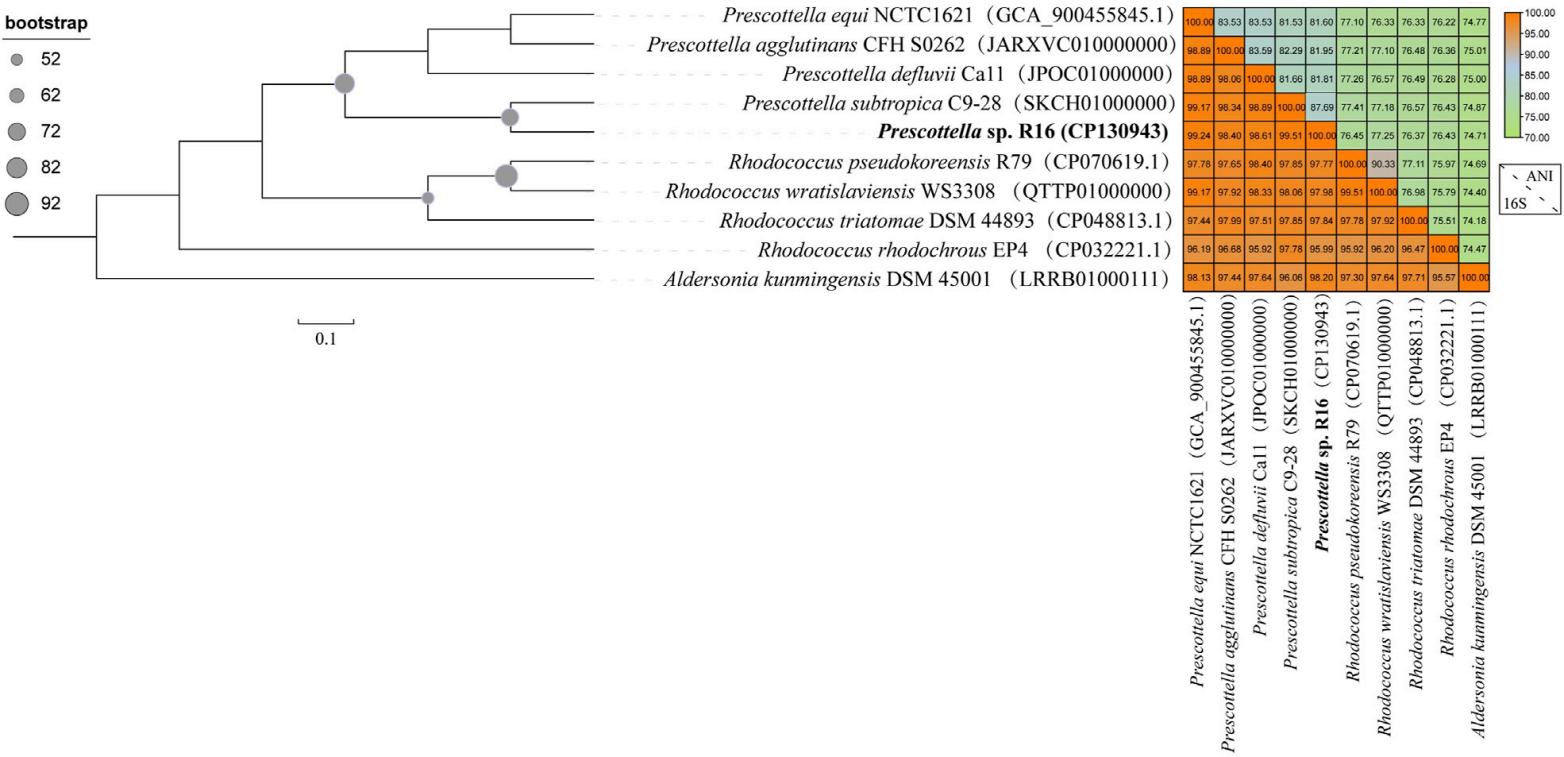
Maximum likelihood tree of 92 concatenated markers and genome similarity heatmap show the affiliations of the *Prescottella* sp. R16(bold font) and eight other strains of the genera *Prescottella* and *Rhodococcus*. The phylogenetic tree was rooted by *Aldersonia* were used as outgroups. The genome sequence of each strain is available from the NCBI database, and the GenBank accession number is shown in parentheses. Bar, 0.1 substitution per nucleotide position.

The taxonomic placement of *Prescottella* sp. has long posed a challenge for taxonomists, with considerable controversy surrounding its classification within the genus *Rhodococcus*. To elucidate its phylogenomic relationships, we employed strain of the *Aldersonia* genus (specifically, *Aldersonia kunmingensis* DSM 45001) as an outgroup for comparative analysis. Additionally, we included five strains from the *Prescottella* genus (*Prescottella equi* DSSKP-R-001, *Prescottella agglutinans* CFH S0262, *Prescottella defluvii* Ca11, *Prescottella subtropica* C9-28, and *Prescottella* sp. R16) along with four strains from the *Rhodococcus* genus (*Rhodococcus wratislaviensis* WS3308, *Rhodococcus pseudokoreensis* R79, *Rhodococcus triatomae* DSM 44893, and *Rhodococcus rhodochrous* EP4) in the construction of a phylogenomic tree based on their genome sequences (Figure 1).

The phylogenomic analysis revealed a distinct topological arrangement within the constructed tree (Figure 1), featuring two primary population clusters alongside the outgroup. Notably, *Prescottella* sp. R16 and four other *Prescottella* strains formed a novel evolutionary branch, exhibiting the shortest evolutionary distance in comparison to *Prescottella subtropica* C9-28. Besides, *Prescottella* sp. R16 shows the highest 16S rRNA similarity value (99.51%) with *Prescottella subtropica*. However, the ANI clearly differentiated the R16 strain from each other and from their closest relatives, with values ranging from 74.71% to 87.69% for ANI that below the threshold 95%–96% for species delineation (Chun et al., 2018). Consequently, we propose that *Prescottella* sp. R16 should be classified as a new member within the *Prescottella* genus, further contributing to the understanding of its taxonomic position.

### 3.3 Genome structural analysis and functional annotation

**FIGURE 2:**
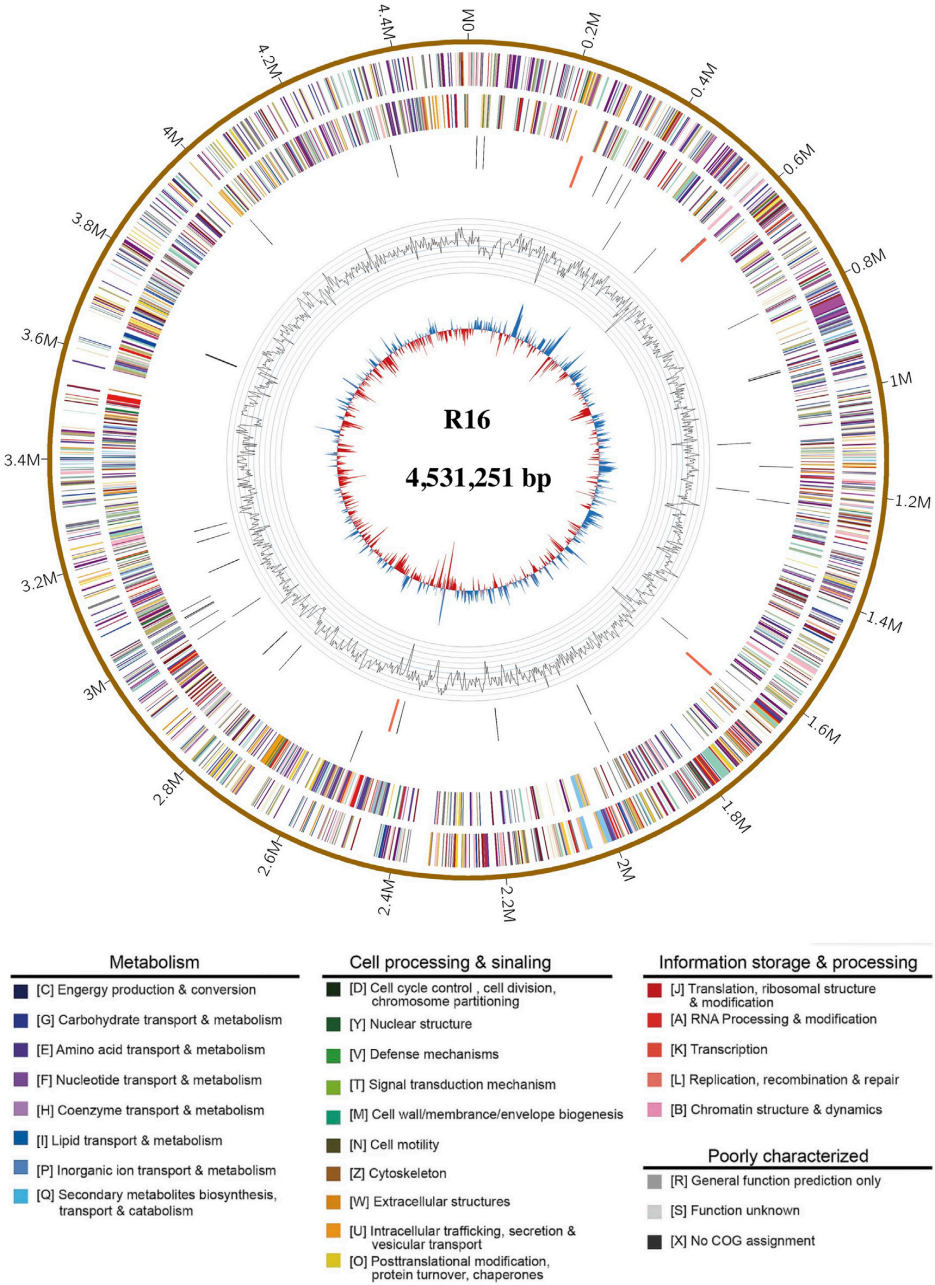
Complete genome map of *Prescottella* sp. R16. Rings from the outside to the center indicate: 1) scale marks for genome size (Mb); 2, 3) protein-coding genes on the forward/reverse strand; 4, 5) tRNA (black) and rRNA (orange) genes on the forward/reverse strand; 6) genomic islands, prediction methods: Integrated (red) and IslandPath-DIMOB (blue); 7) GC content. Protein-coding genes are color coded according to their COG categories (color figure online).

The genome characteristics of strain *Prescottella* sp. R16 and reference strains are presented in Table 1. The complete genome of *Prescottella* sp. R16 contains a circular chromosome of 4,531,251 bp with an average GC content of 68.9%, which consists of 4,208 protein-coding genes, 51 tRNA genes, and 12 rRNA operons. In addition, a plasmid is included, and a total of 12 protein coding sequences (CDS) are annotated using the SwissProt public database (Supplementary Table S1) and visualized according to their physical locations (Supplementary Figure S1). Four CRISPRs and 24 genomic islands are identified in the genome (Supplementary Tables S2, S3). A circular map of the genome was generated in GCView (Stothard and Wishart, 2005) (Figure 2). Based on the IPGA platform, we analyzed the genomes of *Prescottella* sp. R16 and reference strains. Pan-genome statistic visualization is showed on the Supplementary Figure S2 and Supplementary Table S4. When more genomes are analyzed, the larger the pan-genome, the fewer the core genes. Among the 26,435 orthologous gene clusters, only 1,450 (5.48%) were core gene clusters (Supplementary Table S5 and Supplementary Figure S3). Considering the genomic contigs will make an impact on the results, so we independent take the most closed *Prescottella equi* NCTC1621 and *Prescottella equi* NCTC1621 strains that own less contigs for further compare. Among them, there were 695 unique genes belonged to the strain R16 (Supplementary Figure S4). These results provide evidence at the gene level that strain R16 is highly divergent from other species of the genus *Prescottella*.

**TABLE 1:** General features of strain *Prescottella* sp. R16.

Items	Description
MIxS data	
Submitted to insdc	CP130943 (GenBank)
Investigation_type	Bacteria
Project_name	*Prescottella* sp. R16 genome sequencing (PRJNA999030)
Lat_lon	N10°54.7′, E142°19.9′
Depth	6,310
Geo_loc_name	Pacific Ocean: western Pacific
Collection_date	2012-06
Environment (biome)	Marine biome
Environment (feature)	Ocean
Environment(material)	Marine sediment
Project information	
Sequencing quality	Finished
Fold coverage	306×
Seq_meth	PacBio Sequel
Assembly method	HGAP v.4.0
Genome statistics	
Genome size (bp)	4,531,251
Contig GC content (%)	68.9
Is circular	Y
Number of Plasmid	1
Number of genes	4208
Avg. gene length (bp)	985.3
CDS region ratio (%)	91.5

The GO term enrichment analysis of genes provides valuable insights into the potential functions and adaptations of *Prescottella* sp. R16 (Consortium et al., 2004). The presence of rich categories related to catalytic activity, membrane part, and metabolic process highlights their involvement in cellular component, biological process, and molecular function (Supplementary Figure S5A), respectively. The COG analysis results highlight the functional diversity and adaptation strategies of genes in the *Prescottella* sp. R16 genome (Koonin, 2002). The enrichment in categories such as general function prediction only, amino acid transport and metabolism, and lipid transport and metabolism underscore the importance of versatility and resource utilization in the survival and success of these bacteria (Supplementary Figure S5B). Further research focused on characterizing specific genes within these categories can provide a deeper understanding of the molecular mechanisms driving their adaptation to different environments and their potential ecological roles.

### 3.4 Gene clusters related to secondary metabolites

Biosynthetic clusters encoding potential secondary metabolites were identified using AntiSMASH version 5.0 (Blin et al., 2019). A total of 13 putative biosynthetic gene clusters have been discerned (Supplementary Table S6), each presumed to play a pivotal role in the synthesis of diverse secondary metabolites. Notably, five of these clusters bear a substantial degree of homology with previously documented gene clusters. Specifically, these clusters correspond to ε-Poly-L-lysine (100% homology), ectoine (75% homology), heterobactin B/heterobactin S2 (63% homology), isorenieratene (42% homology), and corynecin III/corynecin I/corynecin II (40% homology). Conversely, the remaining eight putative gene clusters demonstrate relatively lower degrees of similarity, with homologies of 30% or less compared to their closest known counterparts. Notably, three of these gene clusters display no discernible similarity to any previously reported gene clusters, thereby suggesting the possibility that they represent novel genetic pathways implicated in the biosynthesis of secondary metabolites.

**FIGURE 3:**
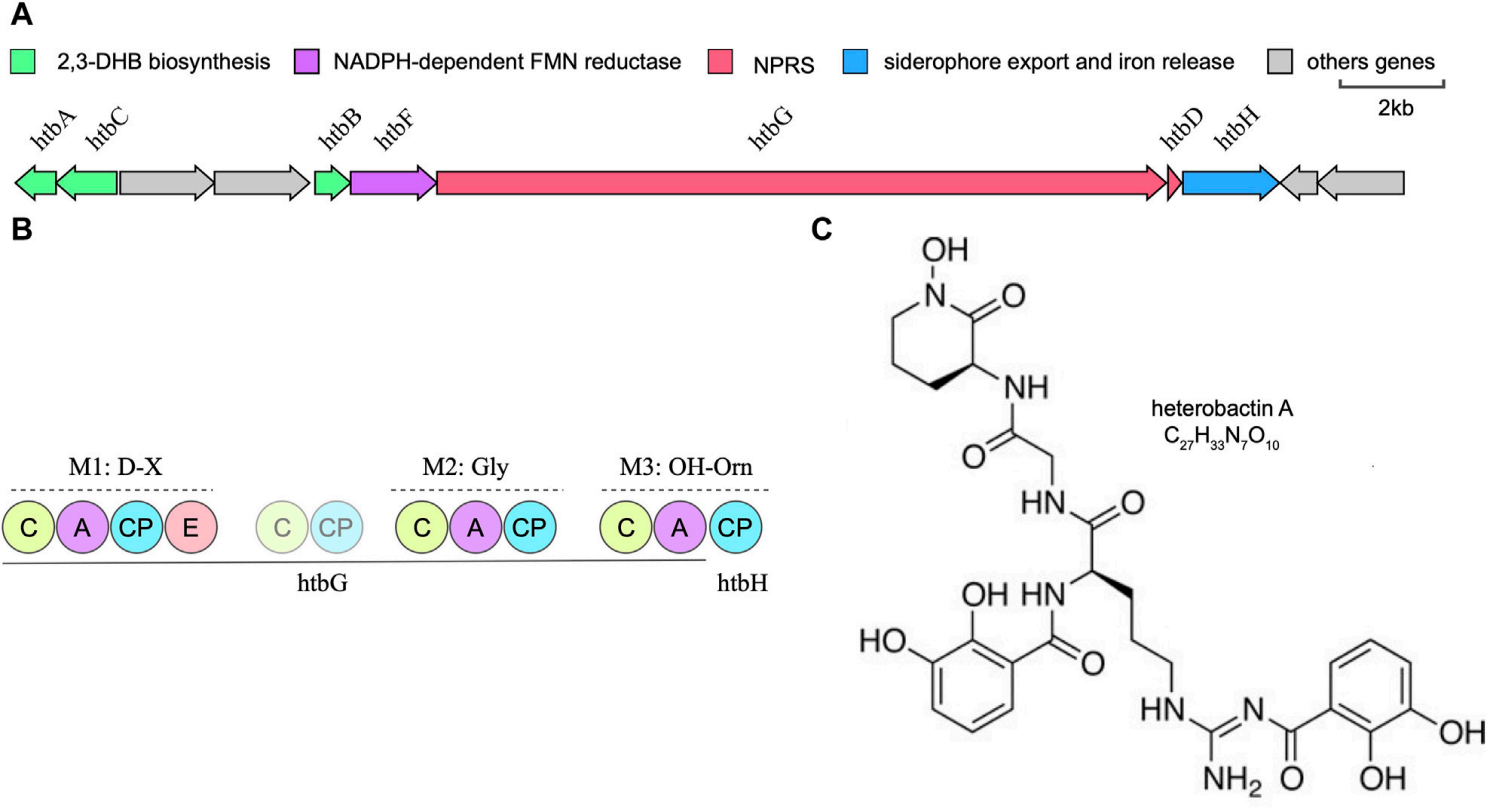
Bioinformatic overview of the heterobactin biosynthetic gene cluster. **(A)** Arrangement of the htb gene cluster in strain *Prescottella* sp. R16. **(B)** Domain organization and the adenylation domain substrate predictions of the NRPSs htbG and htbH. The filled circle means domain in a complete module and the transparent circle mean domain in an incomplete module or outside modules. C:Condensation; A: AMP-binding; CP: PP-binding; E:Epomerization. **(C)** Chemical structure of the heterobatin A.

*Rhodococcus* species have garnered significant attention within the field of microbiology, being the subject of extensive research for their utility as biocatalysts in steroid production and their efficacy as bioremediation agents (Van der Geize and Dijkhuizen, 2004). Whole-genome sequencing data has unveiled the substantial capacity of *Prescottella* for secondary metabolite production. Nonetheless, the isolation of natural products from this genus remains relatively limited. Previous research has elucidated the remarkable chemical diversity inherent in siderophore-related secondary metabolites across the *Rhodococcus* genus (Bosello et al., 2013). Our recent investigations significantly contribute to the broader comprehension of metabolite diversity within *Prescottella*, encompassing remarkable compounds such as heterobactin, capreomycin, corynecin, and several others of note (Figures 3A, B). Moreover, our research has unveiled novel gene clusters that offer a promising avenue for the isolation of previously unidentified siderophores, thus advancing our understanding of their variability within this taxonomic group.

Heterobactin A is a siderophore composed of the tripeptide sequence (N-OH)-L-Orn-Gly-D-Orn-(delta-N-dihydroyxbenzoate) (Figure 3C). The structure of heterobactin A typically includes functional groups that have a strong affinity for iron, such as hydroxamic acid or catechol moieties. These groups form coordination bonds with iron ions, creating stable complexes. The formation of these complexes enhances the solubility of iron in the extracellular environment and allows bacteria to acquire iron for their metabolic needs (Bosello et al., 2013). Iron is a key nutrient for bacterial growth and survival, and studies of heterobactin A’s structure and its relationship with siderophores provides valuable insights into the mechanisms by which bacteria acquire iron.

Here, we report a complete genome assembly of strain *Prescottella* sp. R16 from marine sediments at 6,310 m depth in the western Pacific Ocean based on PacBio technology. The genome size is 4.53Mb (including 1 plasmid), and a total of 4208 coding genes are annotated. Further analysis has confirmed the presence of a secondary metabolite biosynthetic gene cluster in the genome sequence, revealing its potential in lipid transport, metabolism, and amino acid biosynthesis. This research is of great significance for expanding microbial genome resources and will provide valuable insights into understanding microbial diversity, biotechnology, and applications in natural product biosynthesis.

## Data Availability

The datasets presented in this study can be found in online repositories. The names of the repository/repositories and accession number(s) can be found in the article/Supplementary Material.
