# Supplementary material for: Complete genome sequence of a novel Prescottella sp. R16 isolate from deep-sea sediments in the western Pacific
Source: Front Genet. 2024 Mar 14;15:1356956. doi: 10.3389/fgene.2024.1356956 (PMC10972888; doi:10.3389/fgene.2024.1356956)
Supplement: Supplementary file 1 [file DataSheet1.docx]

**Supplementary Figure Captions**

**Figure S1.** Visualization of the protein coding sequence region of the R16 strain plasmid.

**Figure S2.** Statistical visualization of pan-genome gene clusters.

**Figure S3.** Statistical overview of core gene clusters among 10 bacterial genomes.

**Figure S4.** Comparative genomic analysis and visualization between the *Prescottella sp.* R16 genome and two representative bacterial genomes.

**Figure S5.** GO term enrichment analysis and COG analysis of *Prescottella sp.* R16 genome genes.

**Figure S1**

**
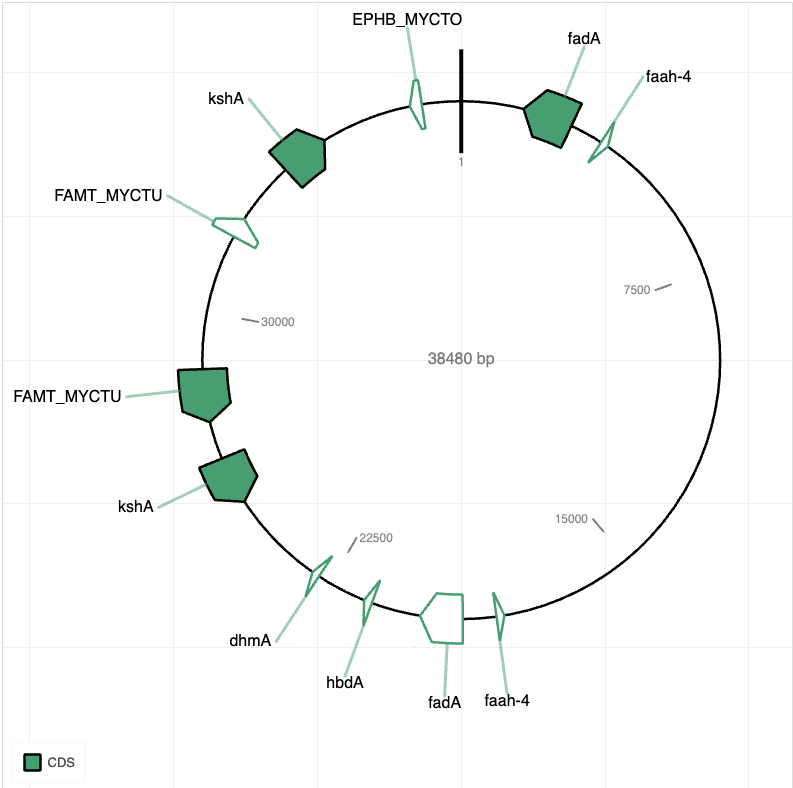
**

**Figure S2**

**Figure S3**

**Figure S4**

**
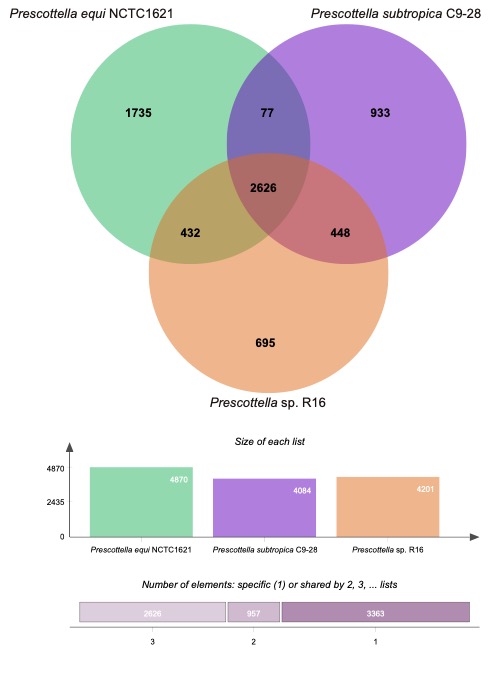
**

**Figure S5**

**
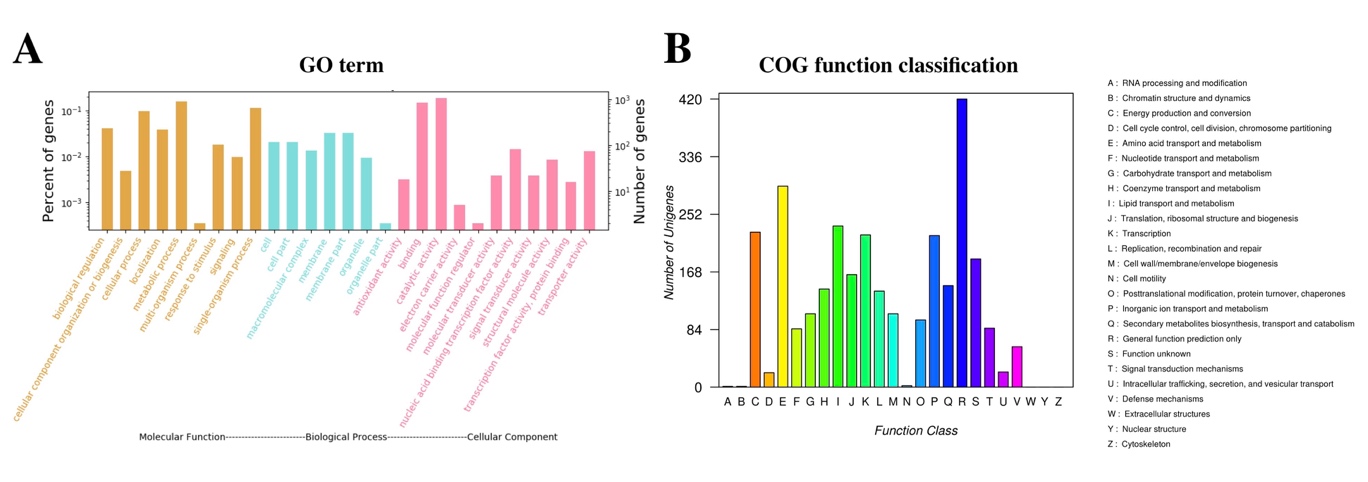
**
